# Functional and Molecular Characterization of New *SPTLC1* Missense Variants in Patients with Hereditary Sensory and Autonomic Neuropathy Type 1 (HSAN1)

**DOI:** 10.3390/genes15060692

**Published:** 2024-05-26

**Authors:** Julie Rochat, André Blavier, Séverine Ruet, Sophie Vasseur, Angela Puma, Béatrice Desnous, Victor Chan, Emilien Delmont, Shahram Attarian, Raul Juntas Morales, Isabelle Quadrio, Léo Vidoni, Nathalie Bonello-Palot, David Cheillan

**Affiliations:** 1 Unité Pathologies Métaboliques, Érythrocytaires et Dépistage Périnatal, Service de Biochimie et Biologie Moléculaire, Centre de Biologie et de Pathologie Est, Hospices Civils de Lyon, 69500 Bron, France; julie.rochat@biomnis.eurofinseu.com (J.R.); severine.ruet@chu-lyon.fr (S.R.); sophie.vasseur@chu-lyon.fr (S.V.); 2 Biokerden, 76130 Mont Saint-Aignan, France; ablavier@biokerden.eu; 3 Service Système Nerveux Périphérique et Muscle, Université Côte d’Azur, Centre Hospitalier Universitaire Nice, 06000 Nice, France; puma.ar@chu-nice.fr; 4 Centre de Référence des Maladies Neuromusculaires de l’Enfant, Hôpital Timone Enfants, Assistance Publique Hôpitaux de Marseille 13915 Marseille, France; beatrice.desnous@ap-hm.fr; 5 Service de Neurologie et Unité Neuro-Vasculaire, Centre Hospitalier de Valence, 26953 Valence, France; vchan@ch-valence.fr; 6 Centre de Référence des Maladies Neuromusculaires et SLA, Hôpital de la Timone, Assistance Publique Hôpitaux de Marseille, 13915 Marseille, France; emilien.delmont@ap-hm.fr (E.D.); shahram.attarian@ap-hm.fr (S.A.); 7 Centre de Reference des Maladies Neuromusculaires Atlantique Occitanie Caraïbe, Département de Neurologie, Centre Hospitalier Universitaire Montpellier, 34295 Montpellier, France; r-juntasmorales@chu-montpellier.fr; 8 Unité Neurogénétique Moléculaire, Service de Biochimie et Biologie Moléculaire, Centre de Biologie et de Pathologie Est, Hospices Civils de Lyon, 69500 Bron, France; isabelle.quadrio@chu-lyon.fr (I.Q.); leo.vidoni@chu-lyon.fr (L.V.); 9 Département de Génétique Médicale, Hôpital Timone Enfants, Assistance Publique Hôpitaux de Marseille, 13915 Marseille, France; nathalie.bonello@ap-hm.fr; 10 Laboratoire Carmen INSERM INRAE, Centre Hospitalier Lyon Sud, 69310 Pierre Bénite, France

**Keywords:** sphingolipids, hereditary neuropathy, *SPTLC1*, molecular genetics, in silico tools

## Abstract

Hereditary sensory and autonomic neuropathy type 1 is an autosomal dominant neuropathy caused by the *SPTLC1* or *SPTLC2* variants. These variants modify the preferred substrate of serine palmitoyl transferase, responsible for the first step of *de novo* sphingolipids synthesis, leading to accumulation of cytotoxic deoxysphingolipids. Diagnosis of HSAN1 is based on clinical symptoms, mainly progressive loss of distal sensory keep, and genetic analysis. **Aim:** Identifying new *SPTLC1* or *SPTLC2* “*gain-of-function*” variants raises the question as to their pathogenicity. This work focused on characterizing six new *SPTLC1* variants using in silico prediction tools, new meta-scores, 3D modeling, and functional testing to establish their pathogenicity. **Methods:** Variants from six patients with HSAN1 were studied. *In silico*, CADD and REVEL scores and the 3D modeling software MITZLI were used to characterize the pathogenic effect of the variants. Functional tests based on plasma sphingolipids quantification (total deoxysphinganine, ceramides, and dihydroceramides) were performed by tandem mass spectrometry. **Results:** In silico predictors did not provide very contrasting results when functional tests discriminated the different variants according to their impact on deoxysphinganine level or canonical sphingolipids synthesis. Two *SPTLC1* variants were newly described as pathogenic: *SPTLC1* NM_006415.4:c.998A>G and NM_006415.4:c.1015G>A. **Discussion:** The combination of the different tools provides arguments to establish the pathogenicity of these new variants. When available, functional testing remains the best option to establish the in vivo impact of a variant. Moreover, the comprehension of metabolic dysregulation offers opportunities to develop new therapeutic strategies for these genetic disorders.

## 1. Introduction

Type 1 hereditary sensory and autonomic neuropathy (HSAN1) is the most frequent autosomal dominant hereditary sensory neuropathy. This disease is characterized by a progressive loss of distal sensory, sometimes leading to chronic ulcerations, necessitating amputation. The sensory impairment comes along with variable autonomic and motor disruption. This phenotype results from several variations of the *SPTLC1* (HSAN1A) and *SPTLC2* (HSAN1C) genes, coding for the two main serine palmitoyl transferase (SPT) subunits [1,2,3]. The SPT enzyme is responsible for the first step of de novo sphingolipid synthesis, which is the condensation of an acyl-CoA with an amino acid, mainly L-serine. The HSAN1 variants result in a gain of function, leading to a switch from L-serine to L-alanine as the preferred substrate of the enzyme. The products of the condensation of L-alanine with an acyl-CoA lack a hydroxyl group, which is essential for the synthesis of complex sphingolipids or for canonical catabolism. These neurotoxic metabolites, accumulating because of this metabolic dead-end, are called deoxysphingolipids [4,5].

SPT is an enzymatic complex located at the membrane of the endoplasmic reticulum. It is responsible for a fundamental step in sphingolipids synthesis and is formed by the association of several protein subunits. The catalytic site is carried by the membrane heterodimer SPTLC1-SPTLC2 or SPTLC3, the two last subunits containing the pyridoxal 5′-phosphate (PLP) binding site required for the reaction. Other minor subunits, like ORMDL3 and ssSPTa/b, are implicated in the regulation of the SPT complex. ORMDL3 exerts feedback on SPT activity, regulated by ceramides plasma levels when ssSPTa/b potentiate the enzymatic activity, and affects the substrate specificity in terms of acyl-CoA [6,7,8,9].

HSAN1 diagnosis is currently based on family history, clinical observations, and genetic analysis using an NGS panel. *SPTLC1* and *SPTCL2* are part of the panel of genes studied for Charcot–Marie–Tooth disease and related disorders. *SPTLC1* and *SPTLC2* variants have been linked to HSAN1; one SPTLC3 variant has also been associated with sensory neuropathy [10]. Molecular analysis provides a strong argument when identifying known and characterized *SPTLC1* or *SPTLC2* variants. On the contrary, due to their specific ”gain-of-function” variations leading to modified substrate affinity, assessing the pathogenicity of the new, undescribed *SPTLC1* or *SPTLC2* variants is challenging.

The in silico prediction of variant pathogenicity is one of the eight evidence categories recommended by the American College of Medical Genetics and American College of Pathologist (ACMG-AMP) guidelines (Supplemental S1). Thus, different tools are available for characterizing variants in terms of physico-chemical, structural, functional, and evolutionary conservation properties. These different tools often produce conflicting interpretations. This leads to classify the variants as variants of unknown significance (VUS). 

To complete these predictions, meta-scores have been developed, which integrate many diverse annotations or individual scoring into a single quantitative score. They combine data on conservation, the frequency of occurrence in a population, and the impact on the physico-chemical properties of the studied protein. Combined Annotation Dependent Depletion (CADD) and Rare Exome Variant Ensemble Learner (REVEL) are two of those meta-scores, with CADD being widely used and REVEL having recently proven to outperform other prediction tools. REVEL only focuses on missense variants, whereas CADD also covers indels and other single-nucleotide variant effect categories such as nonsense or splice-disrupting variants. Moreover, these scores differ according to the databases used to develop them and with the population of variants on which their machine-learning-based scores are trained. These meta-scores help rank the genome variants and could also allow an estimate of the pathogenicity of the newly identified variants in an HSAN1 context [11,12,13].

However, if these meta-scores compile data from several individual tools and integrate different data on conservation, frequency, or physico-chemical properties, they are often based on the same types of information and do not include functional or structural analysis. In the case of a protein complex such as STP, studying the impact of a variant might be more complicated, and individual or meta-scores may not be sufficient to determine the pathogenicity of a variant. The scoring tools and the ACMG-AMP guidelines do not take into account the interactions and regulations within a protein complex. It becomes even more complex when it concerns a “gain of function” variant instead of a “loss of function”. In this case, variations are less deleterious, allowing the maintenance of enzyme activity, even if it is modified. Scoring tools can therefore be faulty. Alternative explorations could provide new insight into the impact of gain-of-function variants on enzymatic activity. 

In this field, in silico protein structural analysis can provide complementing data potentially useful for diagnostic purposes. Indeed, when a protein structure of suitable quality is available, 3D modeling can help with identifying genetic variants that are likely to be deleterious to protein structure and/or function. Identifying the 3D location of the variants, linking it to the functional domains, and studying the interaction disruptions in the direct variant environment provide methods to achieve these conclusions. With SPT being a complex structure, it is essential to study its main subunits as a whole and not each of them separately. Recent studies have clarified the assembly of the human SPT-ORMDL3 complex, thus providing high-quality structures to explore [8,14].

Functional studies are also one of the aspects of the ACMG-AMP guidelines, having a strong impact on the variant classification. When available, functional tests demonstrate the in vivo consequences of genetic variations, instead of the in silico predictions. HSAN1 is characterized by an abnormal metabolism of sphingolipids, mainly an increase of plasma deoxysphingolipids. Therefore, the quantification of deoxysphinganine (dSA) provides a reliable overview of the impact of *SPTLC1* variants on SPT activity and sphingolipid metabolism. 

This work focused on exploring and understanding the molecular and functional consequences of different new *SPTLC1* missense variants. To this end, in silico tools and functional tests based on plasma sphingolipid quantification were used to assess the pathogenicity of six *SPTLC1* variants in patients with HSAN1. 

## 2. Material and Methods

### 2.1. Patients

Six patients with HSAN1, diagnosed based on clinical symptoms and identification of heterozygous *SPTLC1* variants, were studied. These variants were identified using a Charcot–Marie–Tooth panel, sequenced by high-throughput sequencing (List of genes available on Supplemental S2). Their clinical features were collected using a standardized questionnaire, including the different clinical features described in HSAN1 (Table 1). Patient P4, harboring the variant c.992C>T p.Ser331Phe, was previously described and was considered as a “reference” patient [15,16]. For the functional analysis of variants, blood was collected into EDTA tubes, and plasma was immediately isolated by centrifugation (1500× *g*, 10 min, at 4 °C) and stored in aliquots at −20 °C.

The DNA and plasma were prepared and stored by the Biological Resources Center of the Assistance Publique des Hopitaux de Marseille, Marseille, FRANCE (CRB-APHM, certified ISO 20387 and ISO 9001 v2015). The samples were obtained after obtaining written consent from the patient for molecular exploration, post-genomic characterization if necessary, and publication according to the collection declared to the French Ministry of Education, Research, and Innovation (declaration number DC-2008-429), whose use for research purposes was authorized (authorization number AC-2023-5572).

### 2.2. Genetic, Transcript and Structural Data—SPTLC1 (UniProtKB–O15269) Maps to Chromosome 9q22.31

The precise genomic coordinates of GRCh38 are 9:92,031,147-92,115,413. The MANE transcript, composed of 473 amino acids, is NM_006415.4. A protein structure is required for 3D modeling. Two different ones were used: 7k0m, a Protein Data Bank (PBD) structure, and AF-O15269-F1, an AlphaFold-predicted protein structure. The PDB protein structure was recently obtained by electron microscopy and includes the different SPT subunits as well as the PLP location. It was selected because of its high resolution (2.9 Å). However, the SPTLC1 strand of this 7k0m structure does not include the first few amino acids. The predicted AlphaFold structure was necessary to study one of the new variants, even if it only took the SPTLC1 subunit into account.

### 2.3. ACMG-AMP Classification

Classification of the six studied variants was computed through ALAMUT Visual Plus 1.5.1 (Sophia Genetics, 64210 Bidart, France), a genome browser designed to help investigate variations in the human genome. This software suggests an ACMG-AMP classification of the different variants based on different data resources about genome sequence, transcripts, conservation, alignment, variations, and protein domains.

### 2.4. Determination of the Meta Scores

The CADD score (v1.6) was included in the ALAMUT data. CADD uses a machine learning model to evaluate variants’ deleteriousness based on 63 different annotations characterizing conservation and selection, epigenetic information, transcript information such as DNase hypersensitive sites, transcription factor binding, and expression levels. The PHRED-like (−10*log10(rank/total)) scaled C-score ranks a variant relative to all possible substitutions of the human genome. According to CADD recommendations, the pathogenicity threshold was set to a 15 scaled C-score. 

Pre-computed REVEL scores for all possible missense variants are available online through their genomic position. REVEL incorporates individual pathogenicity prediction scores from 13 tools as predictive features: MutPred and Provean scores, functional prediction scores with SIFT, PolyPhen-2, LRT, MutationTaster, MutationAssessor, FATHMM v2.3 and VEST 3.0, and conservations scores from phyloP, phastCons, GERP++, and SiPhy. This scoring method has been specifically trained on recently discovered variants, excluding those previously used to train its constituent tools.

### 2.5. SPTLC1 3D Modeling

Miztli is free online software developed to facilitate access to protein data and characterize variant effects on proteins. It is based on several widely used databases, of which the most important are UniProt and PDBe. The current version β17 is only a prototype and therefore not available for diagnostic purposes. Nevertheless, it allows locating variants in a complex structure and visualizing their consequences on amino acid interactions in their surrounding environment through Mol*, a 3D viewer. The modeling interface highlights the different bonds and interactions between amino acids, including covalent, hydrogen, or cation–pi interactions, and allows switching from the reference to the variant amino acid. This repacking is carried out by FASPR. FASPR not only computes the conformation of the variant amino acid but also adjusts the conformation of other amino acids of the protein. In addition to the p.Ala4Thr variant, requiring the AlphaFold structure, the SPTLC1 variants were studied on the PDB 7k0m structure [17,18]. 

### 2.6. Total Deoxysphinganine (dSA) Quantification

Total dSA was measured using the plasma from the six patients with a method based on those of Penno et al. and Mwinyi et al. [5,19]. Total dSA is the sum of free dSA and sphingoid backbone from hydrolyzed deoxydihydroceramides. Briefly, 500 µL of methanol including the internal standard (d3-1-deoxysphinganine, Avanti Polar Lipids, Alabaster, AL, USA) and 100 µL of ammonia were added to 100 µL of the sonicated plasma sample. Lipids were extracted under constant agitation (1 h, 37 °C). After addition of 500 μL of chloroform and 200 μL of alkaline water, the sample was centrifuged to isolate the organic phase. Acid hydrolysis in 200 µL of methanolic HCl was performed overnight. After neutralization with KOH, 0.5 mL of a methanol-chloroform-KOH solution was added, achieving base hydrolysis. Subsequently, 0.5 mL of chloroform, 0.5 mL of alkaline water, and 100 µL of ammonia were added in this order. Liquid phases were separated by centrifugation, the lower phase was isolated, with the resulting phase washed twice with alkaline water, and then dried under N2. Lipids were resuspended in 500 µL of methanol before analysis by liquid chromatography followed by tandem mass spectrometry (LC-MS/MS). The components were separated on a C8 column (Uptisphère, Interchim© 03100 Montlucon, France), with a precolumn, and analyzed by tandem mass spectrometry on an API 4500 Q-Trap (Sciex Applied Biosystems, Toronto, Ontario, Canada). Molecules of interest were detected through multiple reaction monitoring (MRM), with specific transitions. Quantification was performed with Analyst (1.6.2 version, Sciex Applied Biosystems, Toronto, Ontario, Canada) using an internal standard and a seven-point calibration curve. Data are expressed in nmol/L.

### 2.7. Analyses of Ceramides (Cer) and Dihydroceramides (dhCer) by MS/MS

Analyses of sphingolipids were performed following a method described by Boulet al. [20]. Briefly, a one-phase lipid extraction with chloroform/methanol (1:2) was performed with 10 µL of plasma. Cer (d18:1/17:0) and dhCer (d18:0/13:0-d7) (Avanti polar lipids—Alabaster, AL, USA) were added as internal standards prior to lipid extraction. The lipid-containing phase was evaporated with nitrogen, and dried lipid extracts were stored at −20 °C. Dried lipid extracts were dissolved in chloroform/methanol (1:2) and saponified with 1 M potassium hydroxide in methanol to remove glycerolipids. Samples were then incubated for two hours at 37 °C and neutralized with acetic acid. Samples were purified on an SPE C18 column (Bond Elut) by a double extraction using methanol/H_2_O (1:1), and Cer and dhCer were eluted with chloroform/methanol (1:2). Samples were evaporated with nitrogen, resolubilized in chloroform/methanol (2:1), and analyzed by direct flow injection on a triple-quadrupole mass spectrometer (API 4500 QTRAP MS/MS; Sciex Applied Biosystems, Toronto, Canada) in positive ionization mode using MRM mode. Cer and dhCer were quantified separately with a flow rate of 200 μL/min (analysis time of 3 min). The concentrations of Cer and dhCer were calculated from the ratio of their signal to that of the corresponding internal standard. Total Cer and dhCer corresponded to the sum of the various species.

## 3. Results

The six variants are localized in different domains of *SPTLC1*. c.71A>G (p.His24Arg) is located at the transmembrane domain of the protein; c.451C>T (p.Arg151Cys), c.992C>T (p.Ser331Phe), c.998A>G (p.Gln333Arg), and c.1015G>A (p.Ala339Thr) are located on the intracytoplasmic side of the endoplasmic reticulum membrane, i.e., the aminotransferase domain [6]; c.10G>A (p.Ala4Thr) is an intraluminal variant. No other relevant variant in the other CMT genes was found in the six patients.

### 3.1. In Silico Prediction

*ACMG-AMP classification*—One of the variants, c.992C>T, was previously described as “likely pathogenic” according to the ACMG-AMP classification proposed by ALAMUT and was associated with a more severe phenotype combining early-onset sensory neuropathy and motor deficiency [21,22,23]. The five other variants were uncharacterized and considered as VUSs (Table 2). Their classification was recalculated after the results of in silico prediction and functional analysis.

*Meta-scores*—Scoring was obtained for each of the six variants, through ALAMUT for CADD or directly in the REVEL database. For both CADD and REVEL, the higher the score, the greater the likelihood that the variant is deleterious. 

Considering the pathogenicity threshold established at 15, all six *SPTLC1* variants present a pathogenic C-scaled CADD score. However, one variant is associated with a much lower score, very close to the threshold: c.71A>G. The distinction between the other variants’ C-scaled CADD scores of 24, 25, or even 29 seems less relevant. Based on the CADD scoring tool, out of the six studied variants, only c.71A>G stands out and could have a less deleterious impact (Table 2). 

REVEL scores range from 0 to 1, and, to aid in their interpretation, tables of sensitivity, specificity, positive, and negative likelihood ratio (LR+ and LR−) values are available online. The same as with the CADD score, according to its very low score, REVEL discards the potential pathogenicity of the c.10G>A variant. On the other hand, c.71A>G, c.451C>T, c.992C>T, and c.1015G>A are associated with a REVEL score of 0.75 or higher, suggesting the probable pathogenicity of these variants. The c.998A>G REVEL score was less informative and did not allow the prejudging of the impact of the variant. 

**Figure 1:**
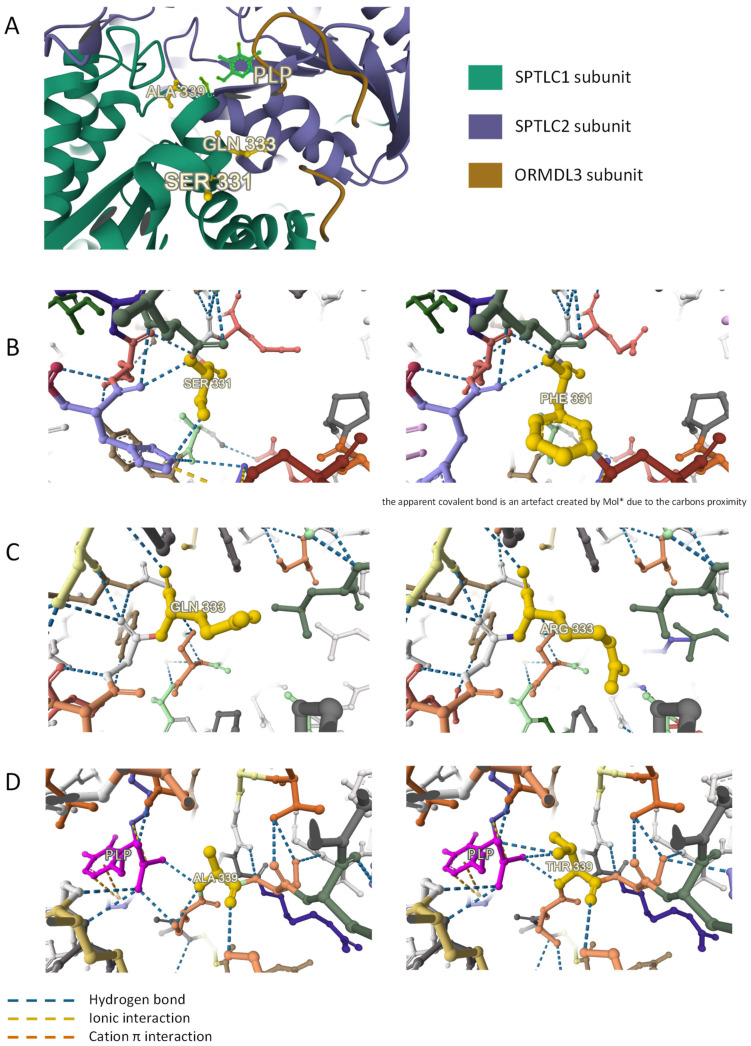
Location of SPTLC1 Ser331, Gln333, and Ala339 residues and Miztli-predicted impact of SPTLC1 p.Ser331Phe, p.Gnl333Arg and p.Ala339Thr variants on the structure and interactions within the SPT enzymatic complex. (**A**) Location of SPTLC1 Ser331, Gln333, and Ala339 residues at the interface between SPTLC1, SPTLC2,and ORMDL3, and near the PLP binding site. (**B**) Impact of SPTLC1 p.Ser331Phe variant. (**C**) Impact of SPTLC1 p.Gln333Arg variant. (**D**) Impact of SPTLC1 p.Ala339Thr variant.

*For 3D modelling*, Miztli allows the study of both the location and close interactions of each variant, as well as the differences between the reference and variant amino-acid on the hydrophobicity scale (Table 2). The active SPT site is formed at the interface between the SPTLC1 and SPTLC2 subunits [23]. Therefore, the variants located at this particular place on the proteins are more likely to have an impact on substrate specificity. Three variants are located at this interface. (Figure 1) p.Ser331Phe presents a large difference in the hydrophobicity scale and results in the loss of a hydrogen bond when p.Ala339Thr interferes directly with the liaison to PLP, the o-enzyme of SPT. Both these variants create steric hindrance in their surrounding environment. Miztli did not indicate any major structural impact of p.Gln333Arg, nevertheless all interactions are not yet taken into account by the software. These three variants are very close to each other and near the PLP binding site, the lysine 379 residue of the SPTLC2 subunit.

The p.Arg151Cys variant is located in the aminotransferase domain but not at the critical SPTLC1–SPTLC2 interface. The large difference on the hydrophobicity scale along with the loss of two hydrogen bonds with the surroundings amino acid could favor the pathogenicity of the variant. 

On the contrary, p.Ala4Thr and p.His24Arg are located in the intra-luminal or the transmembrane domain of SPT. Their impact on intraprotein interactions seems to be non-existent or modest. These findings might be in favor of the benign impact of these variants (Supplemental S3). 

### 3.2. Functional Analysis

**Figure 2:**
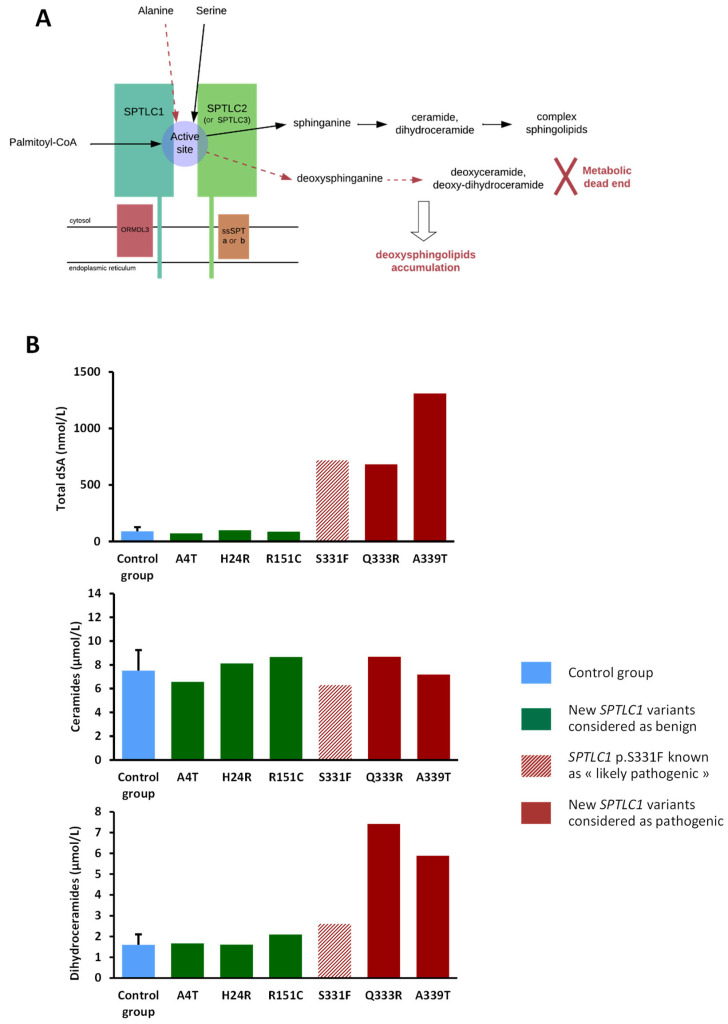
Functional analysis of sphingolipids. (**A**) Simplified SPT structure and role of the enzyme in *de novo* sphingolipid and deoxysphingolipid synthesis. (**B**) Quantification of total dSA, ceramides, and dihydroceramides in plasma samples of patients with *SPTLC1* variants in a context of HSAN1 suspicion, and the clinical–biological conclusion according to the results of the functional analysis.

The first steps of sphingolipids’ metabolism are presented in Figure 2A.

The total dSA level in the plasma was normal for three patients (P1; P2, P3) and a huge elevation for the three others (P4, P5, P6). These results add a functional aspect to the ACMG-AMP classification, allowing the discrimination of most of the variants Accordingly, *SPTLC1* variants associated with high total plasma dSA were considered “Likely pathogenic”; those with levels comparable to controls were considered “Likely benign” (Figure 2B). Consequently, the variants p.Gln33Arg and p.Ala339Thr were upgraded from “VUS” to “likely pathogenic”. Moreover, to determine the metabolic consequences of these *SPTLC1* variants on sphingolipids’ metabolism, total Cer and dhCer were quantified in the plasma (Figure 2B and Supplemental S4 for the details of each molecular species). For the six patients, the plasma level of Cer was comparable to that of the controls, whereas dhCer levels were increased for the three patients harboring pathogenic or likely pathogenic variants, particularly for the two newly described variants.

## 4. Discussion

The recent developments in pan-genomic analysis in the diagnosis of genetic disorders enabled the better characterization of the pathogenic effects of variants identified in genes of interest. *SPTLC1* variants from six patients with HSAN1 were studied using several methods based on in silico prediction tools and functional analysis. One of these variants, p.Ser331Phe, was previously described and associated with an early-onset and severe form of HSAN1, called “S331 syndrome” [21,23]. The other five variants were uncharacterized, and two of them were newly described as pathogenic: p.Gln333Arg and p.Ala339Thr.

Knowing the *SPTLC1* variants’ pathogenicity, clinical presentation was not a significant discriminating parameter: all the studied patients presented the main same symptoms characteristic of HSAN. Nevertheless, age of onset and perforating ulcer of the foot should be considered as hallmarks of severity in patients presenting with a pathological *SPTLC1* variant. Moreover, motor impairment is not usually reported in the typical HSAN phenotype but, in our study, it appeared in 3/3 patients with a confirmed HSAN1A diagnosis.

For each studied variant, meta-scores, modeling, and dSA quantification provided consistent results regarding pathogenicity: no contradicting results were observed, and the significant results were concordant. CADD and REVEL were only able to rule out the pathogenicity of the p.Ala4Thr variant, which presented much lower scores. This result was supported by the location of the variant being totally outside the catalytic site of SPT, as confirmed by the total dSA level, which was comparable to that of the controls. The results obtained with REVEL and CADD were relatively correlated. This observation is consistent with the fact that, despite their differences, meta-scores are based on the same databases. The relationship between the scores obtained and the results of the functional test did not allow the determination of a pathogenicity threshold for *SPTLC1.* Further studies are required to establish gene-specific thresholds, ideally with two levels and a grey area instead of a binary cutoff. Meta-scores are more relevant in helping to prioritize the overwhelming amount of variants identified by exome or genome analysis, rather than in determining the pathogenicity of a specific variant in a gene previously associated with a pathology.

Miztli and 3D modeling divided the variants into two major categories, according to their location: in the catalytic site or outside of it. According to the functional analysis results, the location of the variants seems to matter even more than the local disruption created by the change in amino acids. The two patients presenting a variant located in the intraluminal or transmembrane domain presented a normal total plasma dSA level. Variants at the SPTLC1–SPTLC2 interface were associated with elevated total dSA, consistent with the pathogenic effect of the variant. The p.Arg151Cys variant was indeed localized in the aminotransferase domain but not at the heterodimer interface, which is the precise location of the enzymatic reaction. This slight difference in location might explain the difference in the effect on substrate specificity. In the context of new *SPTLC1* variants potentially associated with HSAN1, studying their location, especially near the SPTLC1–SPTLC2 interface, which is responsible for the catalytic activity, provides a first idea on variant pathogenicity. Nevertheless, functional analysis by quantifying plasma total dSA remains essential to make a definite diagnosis. 

Miztli is a tool under continuous development, with new functionalities and access to complementary databases being added. Access to SWISS-MODEL structural models and Dynamut 2 stability prediction was recently added. Miztli has promising features and an ergonomic interface, and enables the study of interactions and variants’ impacts within protein complexes. This allows a rapid and comprehensive approach to protein 3D modeling, providing an indispensable tool for completing and deepening the in silico prediction of variant pathogenicity. It should not be overlooked that, in this case, recent studies on the SPT complex have provided a high-quality PBD structure, including the different subunits and the coenzyme PLP. However, the availability of the protein structure and its quality are two parameters that can hinder modeling work [24,25]. 

The difference in free energy (ΔΔG) between wild-type and variant structures is increasingly being used for characterizing variant pathogenicity. It provides a numerical assessment of the variant structure’s stability, but the lack of cut off value complicates its interpretation [26]. With “gain of function” variants in a protein complex, ΔΔG does not seem to be a relevant parameter. In the context of HSAN1, pathogenic *SPTLC1* variants increase deoxysphingolipid synthesis and modulate SPT enzymatic activities rather than modify the level of protein expression. Moreover, we hypothesize that the variants probably do not alter enzyme stability. The structural impact or functional impact of the variants would help to more accurately infer pathogenicity. 

In a more general sense, despite the development of numerous prediction tools, the interpretation and classification of novel missense variants remain challenges for diagnostic laboratories. It is important to rely on data from the literature and to combine the different data available: the frequency in the general population, conservation, physico-chemical impact, and 3D modeling. When available, functional testing remains the best option for establishing the in vivo impact of variants and therefore their pathogenicity.

Total dSA quantification enabled our study of the in vivo impact of *SPTLC1* variants on sphingolipids synthesis and particularly on deoxysphingolipids’ accumulation. The elevated total dSA levels of the patient presenting the p.Ser331Phe variant, consistent with the literature, validated the relevance of this functional analysis [22]. Total dSA quantification was the final aspect supporting the *SPTLC1* variants ‘pathogenicity. It allowed us to discriminate the new, undescribed variants as “Likely benign” or “Likely Pathogenic” according to the ACMG-AMP classification by adding a functional component. Moreover, the dysregulation of dihydroceramides in the plasma encountered for the two pathogenic variants occurring around the PLP binding site (p.Gln333Arg and p.Ala339Thr) should be considered as a hallmark of a more severe HSAN phenotype, which can be complicated by motor impairment, as described for the p.Ser331Phe variant [23]. Indeed, HSAN1 is not the only disease associated with dominant *SPTLC1* variants. Recently, several variants of this gene have been associated with the juvenile form of amyotrophic lateral sclerosis. These *SPTLC1* variants cause an increase in physiological sphingolipids through the loss of the negative feedback of the ORMDL3 subunit. Indeed, the *SPTLC1* variants associated with SLA are not located near the active site of the SPT enzyme but near the ORMDL3-interacting transmembrane domain [27,28]. Depending on their location, *SPTLC1* variants can therefore be associated with a clinical picture of motor or sensitive neuropathy. Scoring tools and other common in silico pathogenicity predictors are not sufficient to predict the pathogenicity of a variant that could be associated with diverse diseases. 

However, our study has some limitations. First of all, our approach combining in silico tools, 3D modeling, and functional testing could not be applied to a patient with HSAN1 harboring the most classical *SPTLC1* variant, c.399T>G (p.Cys133Trp). So far, in a French cohort of patients with HSAN, no patient was identified with this variant, but we decided to include a patient with another frequent variant c.992C>T (p.Ser331Phe) who was previously described, to use as a “reference” patient [15]. The number of patients in our work should be considered another limitation. This was mainly due to the low frequency of HSAN1, which could be considered a rare disorder [29]. It would be of interest to extend our work to other patients with other variants to verify our conclusions. Finally, our functional sphingolipid analysis provided some interesting results, but it may be relevant to extend these metabolic investigations to other classes of complex sphingolipids, as recently proposed by Lone et al. [30].

To conclude, we demonstrated the value of using different tools to characterize new variants as well as the importance to of understand the pathophysiologic consequences of these variants. For HSAN1, sphingolipid profiling in plasma led us to develop new biomarkers useful for its diagnosis and to propose therapeutic solutions such as L-serine supplementation; in the future, our results can be used to develop new strategies to regulate sphingolipids metabolism.

## Figures and Tables

**Table 1:** Clinical presentation according to HSAN1-oriented questionnaire.

Patient	Sex	Age of Onset	Familial History	Neurological Impairment										Other Impairments
				Sensitive						Motor				
				Sensitive Impairment	Lower Limb	Upper Limb	Distal Sensory Loss	Stabbing Pain	Paresthesia	Motor Impairment	Lower Limb	Upper Limb	Muscle Atrophy	
1	F	59	Sporadic	+	+	+	+	−	+	−	−	−	−	/
2	F	39	Mother affected	+	+	−	+	+	+	+	+	−	−	/
3	F	36	Sporadic	+	+	+	+	+	+	+	−	+	+	/
4	F	Childhood	Sporadic	+	+	−	+	−	−	+	+	−	+	Perforating ulcer of the foot
5	M	Childhood	Mother affected	+	+	−	+	+	−	+	+	+	−	/
6	F	Childhood	Daughter affected	+	+	+	+	−	−	+	+	−	+	Perforating ulcer of the foot

**Table 2:** ACMG-AMP classifications, in silico prediction, and functional analysis of the six SPTLC1 variants.

Patient	Gene	Genetic Variant	Protein Variant	ALAMUT ACMG-AMP Classification	CADD (PHRED-like) Score	REVEL Score	3D Modelling Observations		Total dSA (nmol/L) Normal Value < 165 nmol/L	ACMG-AMP Classification Updated with Functional Data
							Location	Interactions		
1	*SPTLC1*	c.10G>A	p.Ala4Thr	VUS (PM2, BP4)	15.73	0.101	intra-luminal α-helix	No significant interaction modification	71.5	Likely Benign
2	*SPTLC1*	c.71A>G	p.His24Arg	VUS (PM2, BP4)	**24.5**	**0.805**	Transmembrane domain	Loss of hydrogen bond with Glu28 of the SPTLC1 subunit Steric hindrance with ORMDL3	100	Likely Benign
3	*SPTLC1*	c.451C>T	p.Arg151Cys	VUS (PM1, PM2, PP3, BP4)	**25.1**	**0.759**	Aminotransferase domain	Loss of ionic bond with Glu357 of the SPTLC1 subunit Loss of hydrogen bond with Asn354 and Glu357 of the SPTLC1 subunit Gain of hydrogen bond with Asp147 of the SPTLC1 subunit	87.3	Likely Benign
4	*SPTLC1*	c.992C>T	p.Ser331Phe	Likely Pathogenic (PM1, PM2, PP3, PP5, BP4)	**25.1**	**0.835**	Aminotransferase domain, interface between SPTLC1/SPTLC2/PLP	Steric hindrance with ORMDL3 and SPTLC2 Loss of hydrogen bond with His327 of the SPTLC1 subunit	**717**	Pathogenic
5	*SPTLC1*	c.998A>G	p.Gln333Arg	VUS (PM1, PM2, BP4)	**24.5**	0.504	Aminotransferase domain, interface between SPTLC1/SPTLC2/PLP	No significant interaction modification	**682**	Likely Pathogenic
6	*SPTLC1*	c.1015G>A	p.Ala339Thr	VUS (PM1, PM2, BP4)	**29.1**	**0.844**	Aminotransferase domain, interface between SPTLC1/SPTLC2/PLP	Steric hindrance with SPTLC2 and PLP Loss of hydrogen bond to Ser338 of the same subunit Gain of two hydrogen bonds to the PLP	**1310**	Likely Pathogenic

CADD and REVEL scores and total dSA quantification displayed in bold letters are above the established pathological threshold according to the ACMG-AMP classification; the acronym of each criterion is a composite of P (pathogenic) or B (benign) and the graded strength level: M (moderate), P (supporting), followed by a numerical identifier denoting different types of information. PM1 = mutational hotspot; PM2 = absent from controls; PP3 = computational evidence; PP5 = supporting evidence; BP4 = computational evidence.

## Data Availability

The data presented in this study are available on request from the corresponding author due to ethical reasons.

## Supplementary Materials

The following supporting information can be downloaded at: https://www.mdpi.com/article/10.3390/genes15060692/s1, Supplemental S1: List of abbreviations; Supplemental S2: Genes list of the CMT Panel; Supplemental S3: Miztli predicted impact of SPTLC1 p.Ala4Thr, p.His24Arg and p.Arg151Cys variants on structure and interactions within the SPT enzymatic complex; Supplemental S4: Quantification of ceramides and dihydroceramides species in plasma by tandem mass spectrometry
