# Supplementary material for: Functional and Molecular Characterization of New SPTLC1 Missense Variants in Patients with Hereditary Sensory and Autonomic Neuropathy Type 1 (HSAN1)
Source: Genes (Basel). 2024 May 26;15(6):692. doi: 10.3390/genes15060692 (PMC11203308; doi:10.3390/genes15060692)
Supplement: Supplementary file 1 [file genes-15-00692-s001.zip › genes-2964809-supplementary.pdf]

Supplemental S1: List of abbreviations.

- ACMG : American College of Medical Genetics
- AMP : American College of Pathologist
- CADD : Combined Annotation Dependant Depletion
- Cer : ceramides
- dhCer : dihydroceramides
- dSA : deoxysphinganine
- HSAN1 : Hereditary Sensory and Autonomic Neuropathy type 1
- LR : Likelihood Ratios
- ORMDL3 : ORMDL sphingolipid biosynthesis regulator 3
- PLP : pyridoxal 5′-phosphate
- REVEL : Rare Exome Variant Ensemble Learner
- SPT : Serine Palmitoyl Transferase
- SPTLC1 : serine palmitoyltransferase long chain base subunit 1
- SPTLC2 : serine palmitoyltransferase long chain base subunit 2
- ssSPTa/b : serine palmitoyltransferase small subunit A/B
- VUS : Variants of Unknown Significance

Supplemental S2: Genes list of the CMT Panel.

NM	Gene	NM	Gene	NM	Gene
NM_001605	AARS	NM_000166	GJB1	NM_002677	PMP2
NM_015600	ABHD12	NM_021629	GNB4	NM_000304	PMP22
NM_015270	ADCY6	NM_000183	HADHB	NM_007254	PNKP
NM_014629	ARHGEF10	NM_002109	HARS	NM_021619	PRDM12
NM_015915	ATL1	NM_005340	HINT1	NM_002764	PRPS1
NM_015459	ATL3	NM_000188	HK1	NM_020956	PRX
NM_000052	ATP7A	NM_002148	HOXD10	NM_004637	RAB7A
NM_004281	BAG3	NM_001540	HSPB1	NM_001164730	REEP1
NM_001003800	BICD2	NM_006308	HSPB3	NM_001034850	RETREG1
NM_032667	BSCL2	NM_014365	HSPB8	NM_002972	SBF1
NM_152269	C12orf65	NM_002180	IGHMBP2	NM_030962	SBF2
NM_012073	CCT5	NM_001031714	INF2	NM_014139	SCN11A
NM_213720	CHCHD10	NM_005548	KARS	NM_002977	SCN9A
NM_003632	CNTNAP1	NM_001244008	KIF1A	NM_001113491	SEPT9
NM_004373	COX6A1	NM_015074	KIF1B	NM_015046	SETX
NM_004715	CTDP1	NM_004984	KIF5A	NM_024577	SH3TC2
NM_018122	DARS2	NM_004862	LITAF	NM_005866	SIGMAR1
NM_015726	DCAF8	NM_170707	LMNA	NM_133647	SLC12A6
NM_004082	DCTN1	NM_138361	LRSAM1	NM_021815	SLC5A7
NM_006400	DCTN2	NM_004990	MARS	NM_006941	SOX10
NM_032564	DGAT2	NM_030973	MED25	NM_025137	SPG11
NM_018706	DHTKD1	NM_014874	MFN2	NM_006415	SPTLC1
NM_001039550	DNAJB2	NM_022765	MICAL1	NM_004863	SPTLC2
NM_004945	DNM2	NM_000902	MME	NM_003172	SURF1
NM_001130823	DNMT1	NM_014941	MORC2	NM_177402	SYT2
NM_183380	DST	NM_002437	MPV17	NM_006070	TFG
NM_001376	DYNC1H1	NM_000530	MPZ	NM_015271	TRIM2
NM_000399	EGR2	NM_016156	MTMR2	NM_021625	TRPV4
NM_003640	ELP1	NM_001077186	MYH14	NM_000371	TTR
NM_006329	FBLN5	NM_006096	NDRG1	NM_007126	VCP
NM_030793	FBXO38	NM_006158	NEFL	NM_003384	VRK1
NM_139241	FGD4	NM_002506	NGF	NM_018979	WNK1
NM_014845	FIG4	NM_001007792	NTRK1	NM_003680	YARS
NM_002047	GARS	NM_001142386	PDK3		
NM_018972	GDAP1	NM_020631	PLEKHG5		

Supplemental S3: Miztli predicted impact of *SPTLC1* p.Ala4Thr, p.His24Arg and p.Arg151Cys variants on structure and interactions within the SPT enzymatic complex.

A : Predicted impact of *SPTLC1* p.Ala4Thr variant. B : Impact of *SPTLC1* p.His24Arg variant. C : Impact of *SPTLC1* p.Arg151Cys variant.

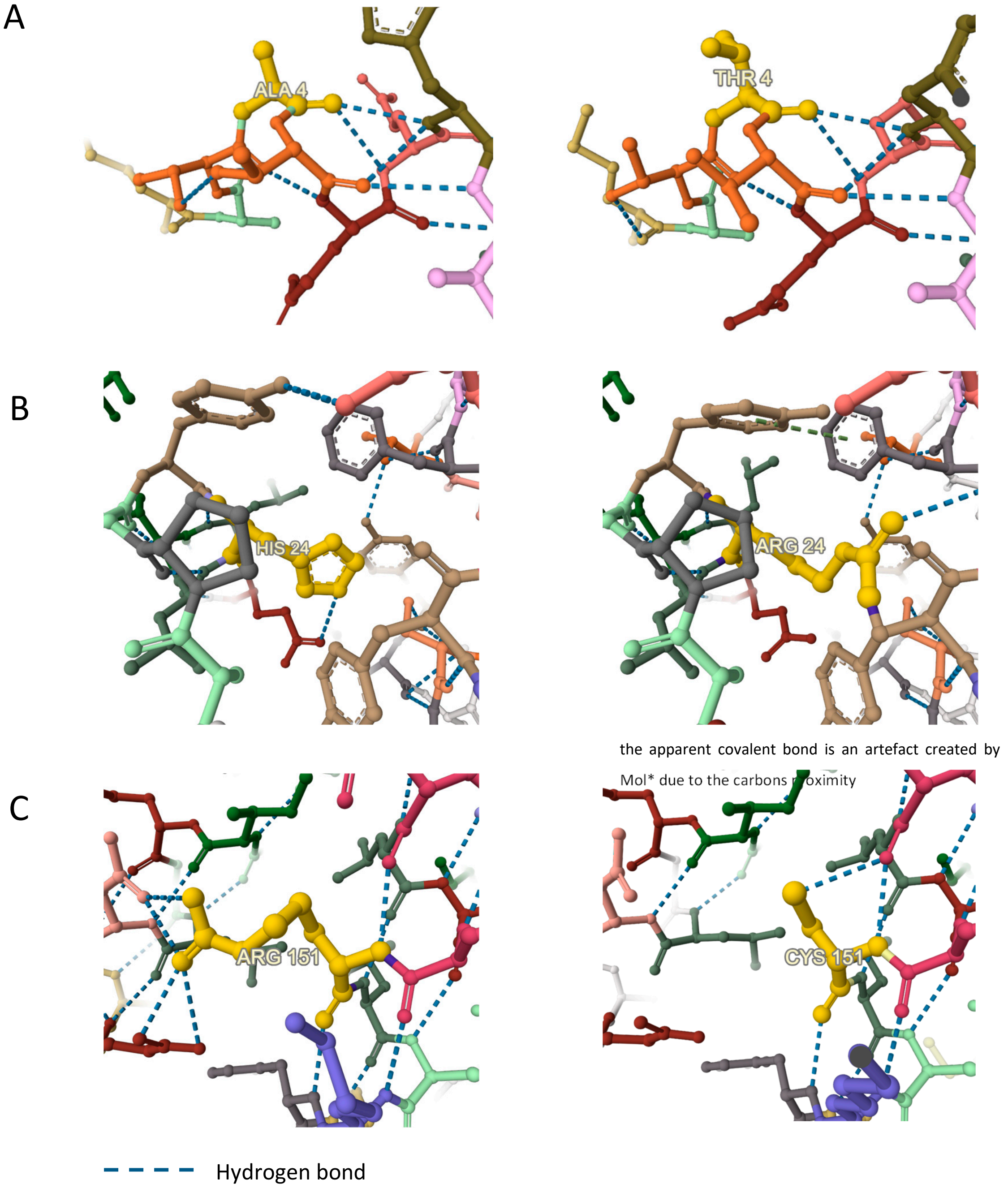

Supplemental S4 : Quantification of ceramides and dihydroceramides species in plasma by tandem mass spectrometry. High levels of Cer of dhCer are shown in red and low levels in light blue.

		Individual ID					
	**Controls**	**P1**<br>p.A4T	**P2**<br>p.H24R	**P3**<br>p.R151C	**P4**<br>p.S331F	**P5**<br>p.Q333R	**P6**<br>p.A339T
**Ceramides** (µmol/L)							
Cer (d18:1/16:0)	1.66 ±0.42	1,24	1,91	1,16	1,51	1,87	3,16
Cer (d18:1/18:0)	0.55 ±0.08	0,53	0,35	0,60	0,24	0,62	0,61
Cer (d18:1/20:0)	0.22 ±0.12	0,42	0,25	0,46	0,28	0,49	0,11
Cer (d18:1/22:0)	1.36 ±0.10	1,17	1,85	1,62	1,29	1,44	0,77
Cer (d18:1/24:0)	3.72±1.26	3,21	3,76	4,82	2,98	4,26	2,54
**Total Cer**	**7.51±1.73**	**6,57**	**8,12**	**8,66**	**6,30**	**8,68**	**7,19**
**Dihydroceramides** (µmol/L)							
dhCer(d18:0/16:0)	0.19 ±0.05	0,27	0,29	0,14	0,16	0,26	0,11
dhCer (d18:0/18:0)	0.07 ±0.02	0,11	0,09	0,14	0,14	1,10	0,88
dhCer (d18:0/20:0)	0.17 ±0.07	0,15	0,11	0,35	0,22	1,54	1,22
dhCer (d18:0/22:0)	0.27 ±0.07	0,20	0,19	0,36	0,86	1,98	1,65
dhCer (d18:0/24:0)	0.90 ±0.23	0,95	0,93	1,11	1,22	2,54	2,03
**Total dhCer**	**1.60±0.50**	**1,67**	**1,61**	**2,10**	**2,60**	**7,42**	**5,89**
